# When a Lung Collapses: A Case of Polymicrobial Empyema Presenting as Massive Hydropneumothorax

**DOI:** 10.7759/cureus.115793

**Published:** 2026-09-04

**Authors:** Eduardo A Irizarry-Rivera, Adriana Suarez Cruz, Julio Torres Rivera

**Affiliations:** 1 Internal Medicine, University of Puerto Rico, Medical Sciences Campus, San Juan, PRI; 2 Pulmonary and Critical Care Medicine, VA (Department of Veterans Affairs) Caribbean Healthcare System, San Juan, PRI; 3 Internal Medicine, VA (Department of Veterans Affairs) Caribbean Healthcare System, San Juan, PRI

**Keywords:** aspiration pneumonia, bronchiectasis, empyema, hydropneumothorax, lung collapse, septic shock

## Abstract

Aspiration-related pneumonia can progress to empyema and massive hydropneumothorax, potentially fatal complications not frequently documented in the literature. Patients with structural lung disease and poor dentition can be particularly vulnerable to severe polymicrobial infections arising from seemingly minor aspiration events. We report the case of a man in his early 70s with chronic bronchiectasis, heart failure with reduced ejection fraction, coronary artery disease status post coronary artery bypass grafting, type 2 diabetes mellitus, chronic kidney disease, and poor dentition who presented with progressive dyspnea and acute hypoxemic respiratory failure. Imaging demonstrated a massive right-sided hydropneumothorax with complete right lung collapse and mediastinal shift. Pleural fluid cultures grew *Streptococcus anginosus* and *Prevotella nigrescens*, consistent with aspiration-related polymicrobial empyema. His hospital course was complicated by septic shock requiring vasopressor support, atrial flutter with rapid ventricular response requiring synchronized cardioversion, and pulseless ventricular tachycardia with successful return of spontaneous circulation. Management included chest tube drainage, broad-spectrum intravenous antibiotics followed by de-escalation, invasive hemodynamic monitoring, and multidisciplinary critical care support. Despite severe multiorgan compromise, the patient ultimately improved and was discharged home on prolonged oral antibiotic therapy. This case illustrates how a seemingly simple aspiration event can progress into catastrophic pleuropulmonary disease with life-threatening cardiopulmonary collapse. Early recognition, prompt source control, and multidisciplinary management were critical to improving the outcome for this patient.

## Introduction

Aspiration pneumonia is caused by inhalation of oropharyngeal or gastric contents into the lower respiratory tract and can present as community-acquired, hospital-acquired, or ventilator-associated pneumonia, depending on the setting in which the aspiration event occurs [1]. Community-acquired pneumonia (CAP) remains a major cause of hospitalization and mortality among older adults, particularly those with underlying cardiopulmonary disease [2]. Although aspiration events are frequently considered benign or self-limited, microaspiration of oral secretions can trigger severe polymicrobial infections that progress rapidly to empyema and respiratory collapse. Elderly patients with structural lung disease, impaired mucociliary clearance, dysphagia, or poor dentition are particularly susceptible to these complications [3,4].

Hydropneumothorax is an uncommon but life-threatening manifestation of pleural infection characterized by the simultaneous presence of air and fluid within the pleural space [5]. Progression of aspiration pneumonia to massive hydropneumothorax, particularly of the severity described in our case, is rarely reported in existing literature. Progressive accumulation may result in complete lung collapse, mediastinal shift, hemodynamic instability, and septic shock [6]. Prompt recognition and source control are essential to prevent rapid clinical deterioration.

We present a case of aspiration-related polymicrobial empyema complicated by massive hydropneumothorax, complete unilateral lung collapse, mixed septic-cardiogenic shock, and cardiac arrest in a patient with chronic bronchiectasis and significant cardiovascular comorbidities. This case highlights how a seemingly minor aspiration event can evolve into catastrophic multisystem disease requiring aggressive multidisciplinary critical care management.

## Case presentation

A man in his early 70s with a medical history significant for chronic bronchiectasis, heart failure with mildly reduced ejection fraction (40-45%), coronary artery disease status post four-vessel coronary artery bypass grafting (approximately 20 years prior), type 2 diabetes mellitus, chronic kidney disease, hyperlipidemia, tobacco use disorder, obesity, and schizophrenia presented to the emergency department with three days of worsening shortness of breath and productive cough.

On presentation, he was tachycardic (116 beats/minute), tachypneic (25 breaths/minute), and hypoxemic, requiring high-flow nasal cannula oxygen support. Physical examination revealed markedly diminished breath sounds throughout the right hemithorax. Initial laboratory studies demonstrated significant leukocytosis (30.5 ×10³/µL) with left shift, elevated procalcitonin (1.49 ng/mL), hyponatremia (128 mEq/L), acute-on-chronic kidney injury (creatinine 2.45 mg/dL), metabolic acidosis, and elevated liver transaminases (Table 1).

**Table 1:** Selected admission laboratory findings

Laboratory Test	Patient Value	Reference Range
White blood cell count	30.5 ×10³/µL	4.5–11.0 ×10³/µL
Procalcitonin	1.49 ng/mL	<0.15 ng/mL
Sodium	128 mEq/L	135–145 mEq/L
Creatinine	2.45 mg/dL	0.7–1.3 mg/dL (male)

Chest radiography demonstrated a large right-sided pleural collection with an air-fluid level and dense opacification of the mid and lower right hemithorax, with loss of aeration of the underlying right lung (Figure 1). Contrast-enhanced computed tomography (CT) of the chest confirmed a massive multilolculated right hydropneumothorax measuring up to 11 cm in maximal dimension with multiple intrapleural gas locules, complete right lung collapse, and mediastinal shift to the left (Figures 2, 3).

**Figure 1:**
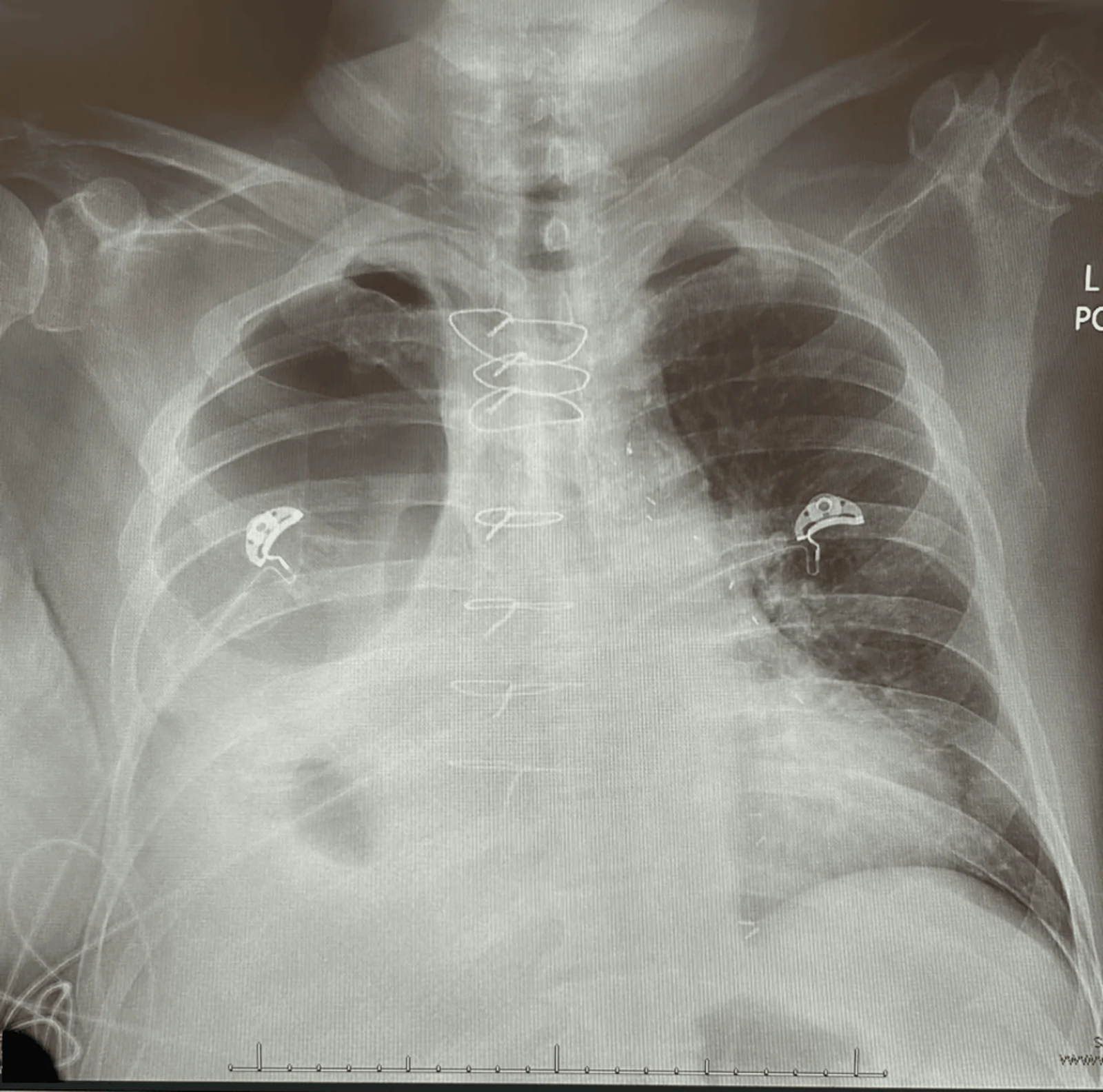
Portable chest radiograph (AP view) on presentation demonstrating a large right multiloculated hydropneumothorax A large right-sided pleural collection with an air–fluid level in the upper right hemithorax and dense opacification of the mid and lower right hemithorax is seen, with loss of aeration of the underlying right lung. The left lung is aerated. Median sternotomy wires and mediastinal surgical clips from prior CABG are present. Surface ECG electrodes overlie both hemithoraces. CABG: coronary artery bypass grafting; AP: anteroposterior

**Figure 2:**
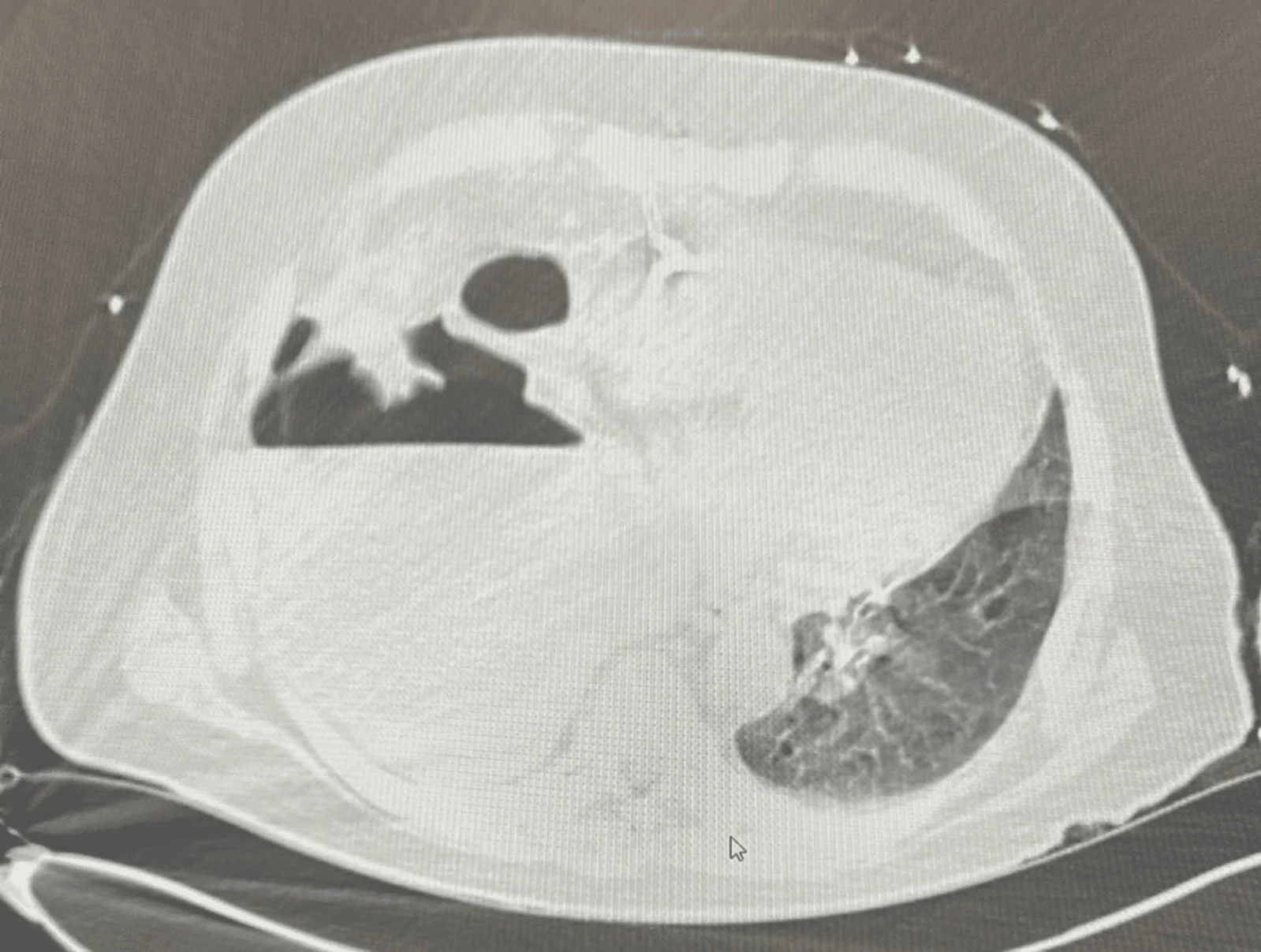
Contrast-enhanced chest CT (axial view) demonstrating a massive multiloculated right hydropneumothorax Hydropneumothorax with multiple intrapleural gas locules separated by soft-tissue septa is seen with near-complete collapse of the right lung, and leftward displacement of the mediastinum. The left lung is aerated, with bronchiectatic change.

**Figure 3:**
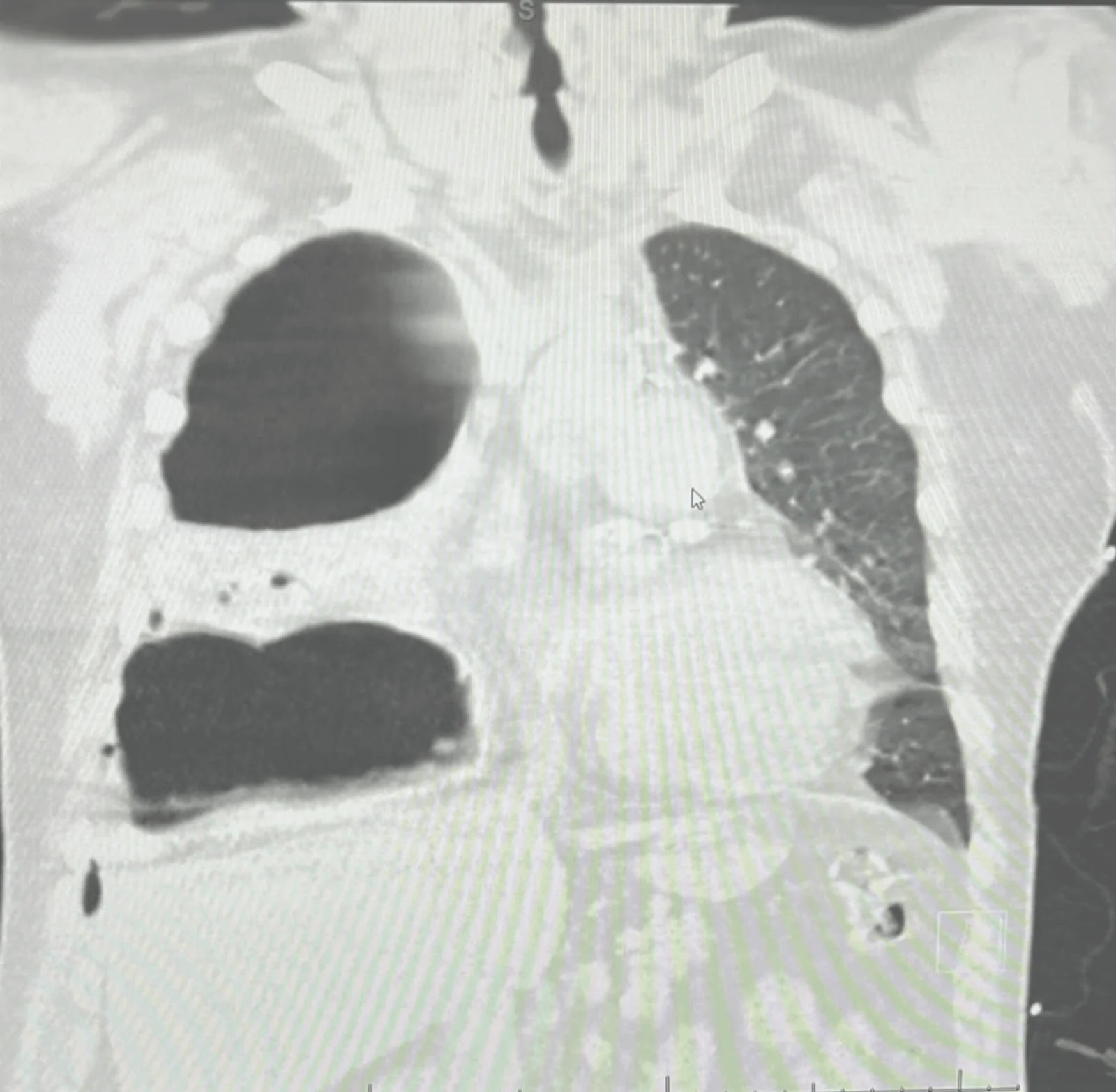
Reformatted contrast-enhanced chest CT (coronal view) demonstrating a multiloculated right hydropneumothorax with lung collapse Multiple loculated gas and fluid collections are seen within the right pleural space with air–fluid levels, near-complete collapse of the right lung, and leftward displacement of the mediastinum. The left lung is aerated for comparison.

He was admitted to the medical intensive care unit for acute hypoxemic respiratory failure secondary to severe pneumonia complicated by hydropneumothorax. Broad-spectrum antimicrobial therapy with vancomycin and piperacillin-tazobactam was initiated. Emergent right-sided chest tube placement yielded a large volume of purulent pleural fluid. Pleural fluid cultures subsequently grew *Streptococcus anginosus* and *Prevotella nigrescens*, consistent with polymicrobial empyema likely secondary to aspiration of oral flora [7,8].

His hospital course was further complicated by septic shock requiring norepinephrine and vasopressin support. Additionally, he developed atrial flutter with rapid ventricular response requiring synchronized cardioversion, followed by a brief episode of pulseless ventricular tachycardia requiring cardiopulmonary resuscitation with successful return of spontaneous circulation. Hemodynamic assessment with Swan-Ganz catheterization demonstrated mixed distributive and cardiogenic shock physiology.

With continued source control, antimicrobial therapy, and supportive intensive care management, the patient gradually improved clinically. Vasopressors were discontinued, invasive monitoring devices were removed, and serial imaging demonstrated re-expansion of the right lung with interval improvement of the pleural collection. Antibiotic therapy was narrowed according to infectious disease recommendations. Further evaluation identified poor dentition as the likely source of aspiration and subsequent polymicrobial infection. The patient was ultimately discharged home in stable condition on prolonged oral antibiotic therapy with close outpatient follow-up involving pulmonology, infectious disease, cardiology, and dental services.

## Discussion

This case illustrates the complications that may arise from aspiration-related pneumonia in older adults. Although aspiration is often perceived as a relatively common and manageable clinical event, the inoculation of anaerobic oral flora into structurally abnormal lungs can precipitate rapidly progressive pleuropulmonary infection with catastrophic complications.

The isolation of *S. anginosus* and *P. nigrescens* strongly supports aspiration of oral secretions as the inciting event. Poor dentition likely contributed to increased oral bacterial burden, while chronic bronchiectasis impaired mucociliary clearance and predisposed the patient to severe infection [3,4,9]. Together, these factors created an ideal environment for polymicrobial empyema formation.

The progression from aspiration pneumonia to massive hydropneumothorax with complete lung collapse represents a rare but life-threatening complication. Accumulation of both pleural air and infected fluid produced a significant mass effect with mediastinal shift, resulting in severe respiratory compromise and hemodynamic instability [5]. He subsequently developed mixed septic and cardiogenic shock physiology, atrial arrhythmias, and cardiac arrest, underscoring the profound systemic effects of advanced pleural infection [6,10,11].

This case also highlights the importance of early imaging and prompt source control. Chest tube drainage, broad-spectrum antimicrobial therapy, and multidisciplinary critical care management were essential to achieving recovery despite the severity of illness. Additionally, recognition of aspiration risk factors-including poor dentition, advanced age, chronic lung disease, and multiple comorbidities-is critical for prevention and early intervention.

Ultimately, this case serves as a reminder that seemingly minor events can evolve into fulminant pleuropulmonary disease with life-threatening cardiopulmonary collapse, particularly in vulnerable patient populations.

## Conclusions

Aspiration-related pneumonia may rapidly progress to polymicrobial empyema, massive hydropneumothorax, and complete lung collapse in patients with structural lung disease and significant medical comorbidities. Early recognition, prompt pleural drainage, appropriate antimicrobial therapy, and coordinated multidisciplinary management are essential to improving outcomes. This case emphasizes how seemingly simple events such as aspiration can trigger catastrophic respiratory and hemodynamic deterioration if not identified and treated promptly.
